# Evaluating a new supported employment internship programme for autistic young adults without intellectual disability

**DOI:** 10.1177/13623613231214834

**Published:** 2023-11-28

**Authors:** Maria Ashworth, Brett Heasman, Laura Crane, Anna Remington

**Affiliations:** 1 Centre for Research in Autism and Education (CRAE), Department of Psychology and Human Development, IOE, UCL’s Faculty of Education and Society, University College London, UK; 2 School of Education, Language & Psychology, York St John University, UK

**Keywords:** autistic young adults, employers, internship, parents, supported employment

## Abstract

Supported employment initiatives have been shown to increase employment rates for autistic people who want to enter the workforce. Despite the success of supported employment initiatives in increasing employment rates, little is known about the first-hand experiences and views of those involved. In the current study, we examined the experiences and views of autistic young adults without intellectual disability (‘interns’), employers and parents regarding a previously unevaluated paid internship scheme that offers tailored support for interns and employers before, during and after the internship, the *Employ Autism network*. In total, 51 participants (19 interns, 22 employers, 10 parents), across eight internships, engaged in semi-structured interviews. Reflexive thematic analysis was used to identify three themes, shared across participant groups: (1) the internship was ‘an invaluable experience’, (2) the internship helped break down autism-specific barriers to employment and (3) stakeholder groups had some unmet expectations. Drawing on this multi-informant qualitative evaluation of a novel supported employment scheme, we derive recommendations of best practice for supported employment initiatives for autistic young adults without intellectual disability.

**Lay abstract:**

Internships designed for autistic people can help them to find employment, but there is little research about the experiences of those involved with internship schemes. To learn more about a new paid internship scheme in the United Kingdom, the Employ Autism network, we interviewed 19 interns, who were autistic young adults without intellectual disability taking part in one of eight different internships. We also interviewed 22 employers (who worked with the interns), and 10 parents (who supported their children in the internship). The interns, employers and parents told us that the Employ Autism network was a valuable experience that helped the interns become more confident. Also, the parents said it helped the interns become more independent. All the groups said the Employ Autism network removed common barriers to employment, and interns and parents said it would help interns get a job in future. Employers and interns said they understood each other better during the internship, and employers said the internship made them think about how their organisations might have accidentally had barriers in place that could stop autistic people getting employed (barriers that they wanted to address in future). However, all groups said some expectations of the internship were not met. These findings suggest the Employ Autism network is helpful for autistic young adults without intellectual disability and employers, but that there are ways that the internships could be improved in future. We discuss the lessons we can learn from the Employ Autism network that might help others who are thinking about setting up, or getting involved in, similar internship schemes.

The transition from education into employment yields poor outcomes for many autistic people (Jones et al., 2022). Despite 77% of autistic people saying that they want to work (National Autistic Society, 2016), autistic people have one of the lowest employment rates in the United Kingdom (29%), even compared with other disability groups (Office for National Statistics, 2022). For autistic people who are in employment, many are underemployed (i.e. in jobs for which they are overqualified) or ‘malemployed’ (i.e. in jobs that are unsuited to the individual’s skillset; Romoser, 2000), which impedes long-term sustained employment (Müller et al., 2003). These negative outcomes have been shown to have a detrimental effect on autistic individuals’ physical and mental health, quality of life and economic wellbeing (Hedley, Uljarević, & Hedley, 2017).

Evidence has shown that supported employment initiatives that include work experience (such as internships) increase employment rates for autistic young people, yet only a minority of the available initiatives have been evaluated (see reviews by Baker-Ericzén et al., 2022; Burgess & Cimera, 2014; Hedley, Uljarević, Cameron, et al., 2017; Hendricks & Wehman, 2009). The few evidence-based work experience initiatives that exist generally include other components of support alongside practical experience. For example, *Project SEARCH plus AS supports* (PS + AS), offers work experience alongside secondary school classroom instruction, behavioural interventions and on-the-job training (Rutkowski et al., 2006; Wehman et al., 2012). The *TEACCH Supported Education Programme* uses an individualised approach for strength-based job coaching and placing for autistic individuals, alongside autism training for employers (Keel et al., 1997). Furthermore, *Prospects* uses support workers to help with job placement, work preparation and liaison with employers (Mawhood & Howlin, 1999). These branded initiatives yield good employment and job retention rates. For example, PS + AS showed 72%–87% in competitive employment up to 40 months post-graduation and 80% job retention (Schall et al., 2015; Wehman et al., 2017, 2020); TEACCH showed 96% employment and 89% retention rates (Keel et al., 1997); and Prospects showed a 68% employment rate (Howlin et al., 2005; Mawhood & Howlin, 1999). Other, small-scale, non-branded work experience trials with similar elements (e.g. job preparation, behavioural intervention, on-the-job training), also report improved employment rates and income (Arikawa et al., 2013; Burt et al., 1991; Hillier et al., 2007).

Despite these encouraging outcomes, there is a need for more evidence-based initiatives (Baker-Ericzén et al., 2022), particularly those that address the limitations of existing supports. For instance, PS + AS is not available for autistic people who are past school-age, despite post-education employment support being sought after by autistic adults (Baldwin et al., 2014). Furthermore, some programmes (e.g. PS + AS) predominantly cater for autistic people with intellectual disability (ID). Yet autistic people without ID can have poorer employment outcomes than their peers with ID (Taylor & Seltzer, 2011), and also report having their support needs overlooked (Crane et al., 2019). While TEACCH can be used by autistic young people with or without ID, it has a limited range of employment opportunities for autistic clients (e.g. clerical, and stocking jobs) which are often part-time and poorly paid. A further limitation is that many of the available work experience initiatives include an element of behavioural intervention (e.g. training regarding social, verbal and non-verbal communication skills). In line with neurodiversity-affirming perspectives and the social model of disability, it may be more worthwhile for work experience initiatives to focus on improving workplace inclusion rather than treating autistic communication differences as ‘deficits’ that should be ‘fixed’ to conform with neurotypical norms (Kapp, 2020; Leadbitter et al., 2021; Oliver, 2013).

Moreover, many evaluations of work experience initiatives have focused on quantitative statistics such as employment rates, job retention levels and income. There is a dearth of literature examining the first-hand experiences of those who take part in these supported internship programmes. In one of the few studies on this topic, Romualdez et al. (2020) interviewed seven autistic young people (17–24 years) with ID and three job coaches involved with PS + AS and gained valuable insight about the positive aspects (e.g. improved self-development for interns and job coaches) and more difficult aspects (e.g. communication challenges) of the experience. Another evaluation of a company-specific internship scheme at Deutsche Bank (DB) UK involved interviewing 16 autistic interns without ID and employers about their experiences of a supported internship scheme (Remington et al., 2022; Remington & Pellicano, 2019). The DB interns reported increased confidence in their ability to fulfil work duties, as well as anxiety related to the stress of working, social demands and miscommunications between intern and colleagues. Employers from DB claimed the internship had mutual benefits for the host organisation by fostering a more inclusive and diverse workplace. Co-workers involved in a qualitative evaluation of an Australian-based employment programme, *Rise@DHHS (Department of Health and Human Services)*, identified similar mutual benefits for the organisation. The autistic employees reported a positive impact on their wellbeing and praised the transparency of the programme’s 3-week work-related training (which was used instead of interviews) that supported a successful transition into the placement (Flower et al., 2019). These in-depth accounts offer valuable insights into the barriers, facilitators and more personal outcomes of supported employment schemes that are missed with quantitative outcome measures (e.g. number of hours worked, income, employment rates).

Bringing together multiple stakeholder perspectives facilitates a more complete understanding of the experience and effectiveness of supported employment initiatives. One perspective rarely explored is parents’ views of work experience schemes. Parents are often deeply involved in their young person’s transition to adulthood due to a lack of support elsewhere, and can feel that it is their responsibility to manage their young person’s support (Chen et al., 2019; Cribb et al., 2019; Hatfield et al., 2017). Indeed, one study that did include parents in a qualitative evaluation of a high-school information and communications technology work-experience placement, *AASQA (Autism Academy for Software Quality Assurance) CoderDojo*, found parents were crucial in preparing their young people for the work experience by providing support with public transport and preparing appropriate work clothes (Lee et al., 2019). The parents also corroborated their young people’s reports of a positive impact on work-related and general confidence, and reported greater hope for their young person’s future. Asking multiple, key stakeholders (i.e. interns, employers and parents) about their views of a work experience supported employment initiative offers a novel, holistic and more comprehensive evaluation.

In the current study, we took a multi-informant approach to conduct the first evaluation of the *Employ Autism network (EAN)*. This novel work experience-based supported employment initiative addresses key gaps in the literature of evidence-based initiatives for autistic young adults. The EAN offers salaried internships at a range of partnered organisations across the United Kingdom to autistic young adults (aged 18+) without ID.^1^ In line with the social model of disability, the EAN offers strength-based support to the interns and employers before, during, and after the internship process that focuses on enhancing environments without any element of behavioural intervention. To gain a holistic understanding of this initiative, we evaluated the first-hand experiences of autistic interns, employers and parents associated with EAN internships. Specifically, we addressed the following research questions:

1. What are interns, employers and parents’ views and experiences of the EAN?
2. Do stakeholders’ views align?

## Method

### The EAN

Following a campaign ran by autistic ‘youth patrons’ to emphasise employment inequalities for autistic people, Ambitious about Autism (AaA) developed the EAN in collaboration their autistic youth council, professionals (combining a range of experience in education, careers guidance, training, as well as lived experience of autism) and employers. The EAN aimed to provide autistic young adults (aged ⩾18 years, without ID) with work experience; building confidence in their transition into employment. The EAN creates zero cost partnerships with organisations in a range of different sectors across the United Kingdom to provide paid internships of varying lengths (from a few weeks to a year), in which interns assist with a range of tasks including (but not limited to) admin, data, and presentation and report writing. Tailored support from staff at AaA is provided to the young adults and the employers before, during and after the internship, including one-to-one help with internship applications, and employer training on understanding autism (see Supplemental Material A for full details of the internship process and the specific support services provided by AaA).

### Participants

Participants were recruited as part of a larger research project tracking and evaluating the EAN, where they indicated whether they would be interested in taking part in an interview once the internship had finished. Those who responded positively were contacted by the research team with information about what the interview would entail, a copy of the interview questions and a link to provide informed consent if they wanted to take part.

A total of 51 participants across 8 organisations were interviewed: 19 interns across six organisations,^2^ 22 employers across seven organisations and 10 parents (eight mothers and two fathers) across three organisations. The internships took place from April to December 2021, ranged from 3 to 15 weeks (*M* = 12 weeks, *SD* = 8.7) and were based in advertising, finance, information technology, recruitment and the public sector (see Table 1 for a full breakdown).

**Table 1.** Participant information.

	Intern (*n* = 19)		Employer (*n* = 22)		Parent (*n* = 10)	
	*n*	%	*n*	%	*n*	%
Age category (years)						
18–25	19	100	1	4.55	0	0.00
26–35	0	0	8	36.36	0	0.00
36–45	0	0	3	13.64	1	10.00
46–55	0	0	10	45.46	2	20.00
56–65	0	0	0	0.00	7	70.00
Gender						
Man (including trans man)	13	68.42	12	54.55	2	20.00
Woman (including trans woman)	6	31.58	10	45.46	8	80.00
Ethnicity						
English/Welsh/Scottish/Northern Irish/British	16	84.21	19	86.36	9	90.00
Irish	1	5.26	0	0.00	0	0.00
Any other White background	1	5.26	1	4.55	0	0.00
Any other mixed/multiple ethnic background	0	0.00	1	4.55	0	0.00
Any other Asian background	0	0.00	1	4.55	0	0.00
Caribbean	1	5.26	0	0.00	1	10.00
Highest level of education*						
GCSEs^a^ (14–16 years)	0	0.00	1	4.55	1	10.00
AS/A-level^b^ (16–18 years)	4	21.05	3	13.64	1	10.00
BTEC^c^ (career-focused qualification for people 14–19 years)	3	15.79	0	0.00	1	10.00
Higher National Diploma (specialist work-related qualification 18+ years)	0	0.00	0	0.00	1	10.00
Foundation degree (vocational qualification 18+ years)	1	5.26	0	0.00	0	0.00
Bachelor’s degree	9	47.37	7	31.82	3	30.00
Post graduate certificate	1	5.26	1	4.55	1	10.00
Post graduate diploma (postgraduate qualification awarded to supplement an original university degree)	0	0.00	1	4.55	1	10.00
Master’s degree	1	5.26	7	31.82	1	10.00
Doctorate	0	0.00	2	9.09	0	0.00
Employment history			Not applicable			
No employment prior to internship	6	31.6				
1–2 previous employers	6	31.6				
3–4 previous employers	5	26.3				
4–5 previous employers	0	0				
5–6 previous employers	1	5.3				
More than 6	1	5.3				
Internship sector						
Advertising agency	0	0	2	9.09	0	0
Banking group	1	5.26	3	13.64	0	0
Computers and information technology company						
Round 1 internship	1	5.26	2	9.09	0	0
Round 2 internship	1	5.26	1	4.55	0	0
Public sector organisation						
Department 1	1	7.14	0	0	1	14.29
Department 2	2	14.29	1	8.33	1	14.29
Department 3	2	14.29	0	0	1	14.29
Department 4	0	0	1	8.33	0	0
Department 5	1	7.14	1	8.33	0	0
Department 6	2	14.29	2	16.67	1	14.29
Department 7	1	7.14	0	0	0	0
Department 8	1	7.14	0	0	2	28.57
Department 9	2	14.29	0	0	0	0
Department 10	1	7.14	1	8.33	0	0
Department 11	0	0	0	0	1	14.29
Department 12	0	0	1	8.33	0	0
Department 13	0	0	1	8.33	0	0
Department 14	1	7.14	4	33.33	0	0
Employment agency	1	5.26	1	4.55	0	0
Information technology company	0	0	0	0	1	10
Recruitment company	1	5.26	1	4.55	2	20

GCSE: General Certificate of Secondary Education; AS/A: Advanced Subsidiary/Advanced level qualifications; BTEC: Business and Technology Education Council.

* Levels of education are listed from the lowest to the highest level.

Interns were autistic, between the ages of 18 and 26 (*M*_age_ = 22.5 years, *SD*_age_ = 2.5) and did not have ID, confirmed by their full-scale IQ composite scores from the matrix reasoning and vocabulary subscales of the Wechsler Abbreviated Scale of Intelligence Second Edition (WASI-II; Wechsler, 2011), which ranged from 84 to 114 (*M* = 101, *SD* = 7.75). The ‘employers’ group included the interns’ line managers, heads of department and members of the interns’ wider teams. The ‘parents’ group were mostly parents of the intern participants in the study, but there were two instances where a parent and employer opted in, but their young person did not. See Table 1 for further details.

### Materials and procedure

Ethical approval was obtained from the Department of Psychology and Human Development at IOE, UCL’s Faculty of Education and Society (no ethics ID numbers are provided for PhD research projects at IOE). Participants took part in remote interviews with the first author shortly after their internship finished (from June 2021 to January 2022). Although each participant group had a different interview schedule (see Supplemental Material B), they followed a similar structure and content: (1) welcome, overview and opportunity to ask questions, (2) previous experience, (3) experience/views of the internship, (4) outlook after the internship and (5) closing comments and thanks. Identical or equivalent questions were included in each group’s interview schedule where possible. The average length of the interviews was 27 m:09 s (*SD* = 8 m:59 s, range = 11 m:14 s–49 m:35 s).

Efforts were made to maximise the inclusiveness and accessibility of participation such as providing interview questions in advance and offering a range of synchronous/asynchronous options for participation. All participants opted to participate synchronously via Zoom.

### Community involvement

Two autistic young adults (an 18-year-old man and a 24-year-old woman) were consulted to review the interview guidelines and schedules and ensure the questions were appropriate, clear and precise. The consultants had previously completed an EAN internship but were not involved in the current research. Feedback led to revisions to introduction and closing sections (to improve clarity), the addition and removal of questions, and editing questions and probes to be more precise and provide sufficient context. This feedback was provided to the first author via email and/or video meetings. One consultant provided feedback via a proxy reporter (their mother) who relayed the feedback via email. In line with the UK’s national advisory group’s guidance for public involvement in health and social care research (INVOLVE guidelines, National Institute for Health Research, 2016), consultants were paid £75 each for the equivalent of 4 h across 1 month.

### Data analysis

Interviews were transcribed verbatim, and transcripts were analysed using NVivo software following Braun and Clarke’s (2006, 2019, 2022) six stages of reflexive thematic analysis. Data were triangulated across multiple respondent groups (interns, employers, and parents) to understand multiple viewpoints of the internship. We examined overall similarities and differences between the views of these groups, rather than comparing specific views within dyads or triads per internship. Participant groups were analysed separately, but during stage three (generating initial themes), a decision was made to report the results from the groups together considering the theme’s overlap, and include relevant details of a group’s specific views within each theme. Data were analysed through a critical-realist framework whereby participants’ reports were taken as their reality but also recognised within a wider influence of social context on perspective. A semantic, inductive approach was used to identify patterns and themes, describing and interpreting the data explicitly at its surface meaning without a predetermined coding frame (Braun & Clarke, 2006; Willig, 2013).

Data analysis was led by the first author, supplemented by regular discussions with the broader research team. Before starting analysis, the first author kept a reflexive journal to acknowledge their inference and influence of the data (Ahern, 1999). None of the authors have direct personal experience of the topic of research study, and all authors view autism from a social model of disability which recognises the role societal barriers play in disabling autistic people, instead of viewing autistic people as inherently ‘impaired’ (Oliver, 2013). In addition, the authors included elements advised by Nowell et al. (2017) to ensure trustworthiness of the data, including prolonged engagement with the data, documenting team debriefing meetings about codes and themes, continued diagramming to determine theme connections, repeated team reviews of themes and theme names to reach consensus, and checking of drafts of the report.

## Results

**Figure 1.**
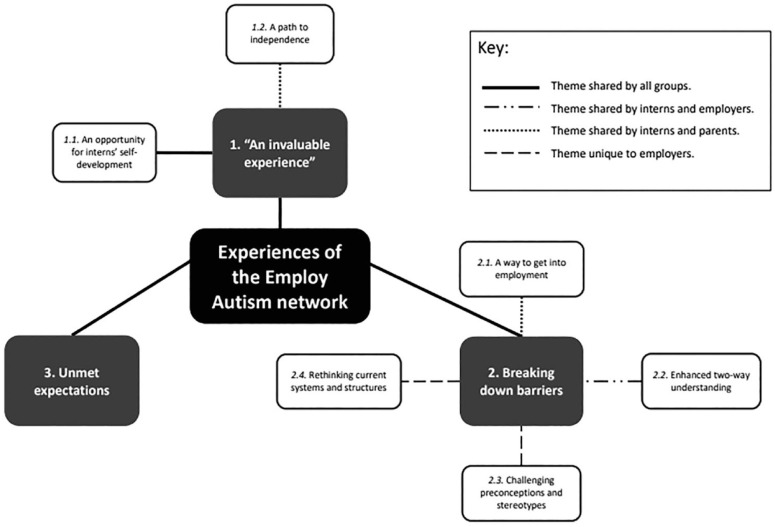
Thematic map.

We identified three themes shared by interns, employers and parents.^3^ Within these themes, we identified six subthemes. Four subthemes were shared between groups, and two were unique to employers (see Figure 1, in addition to Supplemental Material C for further illustrative quotes).

### ‘An invaluable experience’

Interns, employers and parents reported that the internships were a rewarding experience. Specifically, the internships provided *an opportunity for the interns’ self-development* professionally and personally (*subtheme 1.1.*), and *a path to independence* for the interns’ futures (*subtheme 1.2.*).

#### An opportunity for interns’ self-development

Interns felt that the internship improved their confidence in their own capabilities:

I definitely feel more confident in my ability to feel like I can get things done. . . I think I always feel a bit afraid that I’m not capable of things. . . but actually most people can do most jobs and I can definitely do most stuff that is thrown at me. . . delivering what people want from me and expect from me. (I8)

Parents corroborated this improved confidence and highlighted how the internship provided experience of working life that may be crucial for future employment prospects:

Getting used to working with people, people you don’t know very well, dealing with all the small talk. . . understanding how the office works and the idea of commuting and doing a job, and the hours you have to be there for and just that general understanding of how the world goes round. (P8)

Interns attributed improved self-confidence to work being meaningful and fulfilling, and feeling valued by employers:

Now I have got concrete evidence that I can do a decent job, and I have praise from the line managers who both said that if they could they would like to keep me as a permanent employee, which was very flattering to hear. (I13)

Employers echoed this sentiment, describing the interns as a ‘genuine asset’ (E6) to their teams. Employers discussed their efforts to make the internship a positive and rewarding experience where the interns could get ‘solid things to go within a CV’ (E11).

One intern reflected that their experience impacted their self-perception of being autistic: the fact that I’ve come into [organisation’s name] consciously having autism and them consciously trying to help and support me, because of that I feel like I’ve learned a lot about how autism affects me. (I3)

Such experiences led interns to wish they had benefitted from the opportunity earlier in their transition from education to employment, but also considered whether they would have been comfortable disclosing their autism diagnosis at an earlier stage:

I would have liked to have known about AaA earlier in my life, but I can’t say confidently that I would have been comfortable at that point, because, obviously, you have to disclose [an autism diagnosis] that’s the nature of how these programmes go. (I1)

#### A path to independence

Opportunities for self-development fed into a wider concept of independence. Interns discussed how improved confidence helped them take more initiative and ownership of their responsibilities and day-to-day lives, reporting that this fostered greater independence: ‘I eventually started doing things of my own accord. . . I felt that level of independence, I was getting a lot done’ (I17).

Independence was also a key theme for parents, who wanted their young adults to have autonomy but, in some cases, noted ‘I don’t think [my young person] is up to doing it for himself’ (P2). The EAN experience provided parents with reassurance about their young person’s developing independence, and they described how ‘It’s given me for confidence for [their] future’ (P3).

### Breaking down barriers

Interns, employers and parents reported different ways the EAN helped mitigate aspects that often impede employment for autistic people. Some features of the EAN provided *a way to get into employment* (*subtheme 2.1.*), and there was *enhanced two-way understanding* between interns and employers *(subtheme 2.2.)*. Employers reflected on *challenging preconceptions and stereotypes* of autism *(subtheme 2.3.)*, and started to *rethink current systems and structures* in their workplace that might preclude employment for autistic people *(subtheme 2.4.)*.

#### A way to get into employment

Interns highlighted how common barriers to employment were broken down as part of the internship, enabling autistic young adults to get ‘a foot in the door’ (I13).

The accessibility of the application process was valued by interns, especially being able to approach staff at the EAN for support: ‘it meant that, instead of me being given an open question that I don’t know how to answer, it’s interactive and I can ask for clarification, so I know what I’m supposed to be saying’ (I7). There was also recognition of the adaptations made to interviews, and how this offered a smooth process from initial application to successful receipt of an internship place:

It was a tad daunting, but . . . it was a lot smoother and easier [than previous experiences] because I knew that I didn’t have to conceal my autism or to downplay it [in the application process] . . . they were mentioning any changes that needed to be made to the interview, so I said that if I could have a copy of the interview questions beforehand I would be more able to prepare decent answers which reflected me more as a person . . . that support was fantastic. (I13)

Parents acknowledged EAN’s involvement as valuable to help their young adults get into employment:

I just think it’s an absolutely awesome programme . . . I’m glad that [my young person] had the opportunity to be able to have that experience and I think sometimes there does need to be like a conduit or a third party to be able to assist bridging that gap. Working in [organisation’s name], they may not openly take individuals for work experience . . . having an organisation like [the EAN] be able to assist . . . is great. (P5)

#### Enhanced two-way understanding

Interns reported that the communication between themselves, their employers and their colleagues ‘turned out to be quite effective’ (I8). Employers were perceived as friendly and inclusive (‘everyone there was very, very helpful and welcoming to me and very patient with me, because sometimes I take a bit of time to understand things’; I19), which was reportedly surprising: ‘everyone was really nice. I didn’t expect it’ (I10). The interns appreciated the internship being autism-specific (‘I’m in a job where everyone there is informed about (the fact that I’m autistic), and I do find it nice’; I19), and reflected that the training and support employers received may have led them to be more accommodating. Such support was seen as facilitative to other learning opportunities, including the social aspects of working life:

The number one positive is the people, connecting with people, having useful conversations with people or sharing different perspectives . . . I think that’s been useful just being able to encounter all of that. (I9)

The employers felt that the EAN internship enabled both parties to gain something valuable, noting that the interns offered a ‘different perspective’ (E5) in the workplace: ‘having somebody who is autistic who thinks through things in a slightly different way is really, really valuable’ (E19). Relatedly, employers explained that the internship afforded a wider learning opportunity in encouraging employees to be more understanding and accepting of difference:

It’s reminded me how similar and yet different we all are, and it’s reminded me about some simple things that you just tend to forget about sensitive issues and how they come up for people . . . and how bad we are as a society of needing people to present in a cookie cutter frame. (E11)

In one instance, this culture of inclusivity led another colleague to disclose their own neurodivergence.

#### Challenging misconceptions and stereotypes

Prior to the internship, the employers described apprehension about hosting an autistic intern. Employers ‘didn’t know what to expect’ (E3) from the internship (e.g. would they be difficult to manage and support?), and/or had a ‘preconception of what an autistic person might be like’ (E18; e.g. would analytical tasks suit them best?). The training received was ‘really good at debunking a lot of myths . . . the ones that are sort of more positive like, autistic people are really clever or everyone is on the spectrum’ (E17). Yet the employers’ noted that some misconceptions about autism persevered, and could lead others to ‘underestimate [the interns] in terms of [their] capability to do tasks’ (E16).

I felt some of my colleagues maybe had lower expectations and were overly hesitant . . . if [they] hadn’t told me [they] were autistic, I wouldn’t have known and we would have challenged [them]. So why shouldn’t we challenge [them] just because [they’re] autistic? (E13)

However, the employers explained that working closely and directly with the autistic young adults on the internship helped dispel preconceptions.

#### Rethinking current systems and structures

Following the internship experience, employers questioned why their intern had previously had such difficulty finding work: ‘It struck me as why does this individual need this internship? [They] should have already been employed. [They’re] great’. (E13). Employers reflected on their current practice as they recognised some of the barriers to employment the interns could face: ‘you realise that the organisation has created a lot of challenges and problems, so it can be a bit humbling’ (E2). For example, the employers acknowledged that the recruitment processes for their organisations could be disadvantageous for autistic applicants:

[our assessment centre] isn’t set up to cater for somebody on the spectrum because it’s just expected that somebody’s going to go and spend a whole day in a group doing different tasks . . . from talking to [the intern] you could tell visibly [they] wouldn’t like that . . . [they] would be very quiet and it would probably look bad from a behavioural point of view. (E18)

In addition, the employers highlighted the importance of making workplace adjustments and their wider benefits for the workplace (‘there isn’t a huge amount of changes that need to be made . . . just some really small changes that will actually benefit your communication style more generally’; E7) and highlighted that inclusive practice should ‘become part of your work culture and behaviour’ (E4) to be sustained long term.

The employers admitted ‘historically, organisations haven’t got this content right’ (E2) so were ‘heavily invested’ (E1) in the internship to ensure it was successful. As such, employers advised to ‘get the support you need’ (E2) before welcoming an autistic person to the organisation (‘I think if [the intern] joined without the programme, it would have been really hard for [them] and it would have been really hard for us’; E3), and said the tailored support provided by the EAN ‘gave [the employers] the confidence to work with [the intern]’ (E2).

### Unmet expectations

There were some unmet expectations, due to a mismatch in interns’ and employers’ expectations regarding support, skills matching and outcomes of the internship.

First, the interns felt there were some misjudgements from employers related to how much support they needed and when, which was reported to be unintentionally patronising:

Once or twice, I encountered a situation where someone would want to be very nice to me, but the way they would phrase it would come off as a bit patronising. . . they were like, your brain is a lot more analytical than mine, and that made me feel really awkward because you’re having to live up to the expectations of what someone might think your neurodiversity is . . . I don’t feel like that comes from a place of being intentionally patronised, that’s just phrasing if you want to be welcoming and complimentary and it might come across wrong. (I19)

Second, while some interns got what they wanted from the internship ( ‘I had objectives to build my confidence and get work experience. . . I think I gained all of that’; I6), other interns discussed having different hopes of what their role would be, and highlighted some mismatching of skills to work: ‘I was told I was going to be put into a role that fit my skill set in some way, but it ended up being a lot of correspondence and writing tasks which is stuff I always found difficult’ (I7). Relatedly, the employers said they wanted more information about their specific intern’s skills ahead of the internship so they could provide appropriate and timely support, plan and match suitable tasks to their skillset: ‘[the candidate profile should] not just list [their] hobbies and [their] degree, but what [they] felt [they] would be able to do as an intern. . . [that] would have helped us identify what tasks would have suited [them]’ (E13).

Finally, other interns wanted more clarity on the outcome of the internship: ‘while [the internship] has prepared me a bit, I still don’t know if I can actually get a job that I feel is appropriate to my skills’ (I7). Indeed, parents questioned whether the internship afforded the interns too much support and gave ‘a slightly unrealistic view of what the real world of work is like’ (P6).

## Discussion

Existing literature on supported employment schemes has focused on quantitative outcomes such as employment rates and income (see reviews by Baker-Ericzén et al., 2022 and Hedley, Uljarević, Cameron, et al., 2017). Our findings highlight the first-hand impact that work experience initiatives have on the people involved. Specifically, interns and parents in our study reported that the EAN provided an opportunity for the interns’ self-development, with improved self-confidence in personal and work-related capabilities. These findings echo similar reports of interns’ enhanced self-efficacy on the PS + AS (Romualdez et al., 2020), DB UK (Remington et al., 2022; Remington & Pellicano, 2019), and AASQA Coder Dojo work experience schemes (Lee et al., 2019), further cementing work experience as an important facilitator of self-development for autistic adults who want to work.

Crucially, employers reported that the experience challenged their preconceptions about autism and made them rethink their organisation’s current systems and structures. Previous research has shown that neurotypical people’s biases about autistic people are often based on minimal information, which negatively affects their first impressions of autistic individuals (Sasson et al., 2017). However, knowing an individual is autistic and having a better understanding of autism can improve such judgements and perceptions (Morrison et al., 2019; Sasson & Morrison, 2019). The current findings may reflect this narrative in that employers reported initial apprehension about the internship related to a lack of understanding about autism. Similarly, in line with the *contact hypothesis* (Turnock et al., 2022), interactions with the interns via work experience may have increased employers’ understanding to develop new perceptions of autistic people, and realise and reconsider the systemic barriers in their workplaces. As such, work experience in real-world settings may be an important component of supported employment initiatives for autistic people without ID.

Many interns viewed being openly autistic on the EAN internships as positive. Interns felt that the autism-specific nature of the internship provided a rare but valuable opportunity to be more authentically themselves, and bolster self-esteem by exploring their capabilities. However, it should be noted that the interns in the present study may have already held a certain level of confidence in their autistic identity, to be involved in an autism-specific internship. This confidence may not hold true for the wider autistic population due to complex, personal choices around disclosure. For example, many autistic people choose not to disclose their autism diagnosis to employers to avoid damaging stereotypes and/or discrimination related to being autistic (Romualdez, Heasman, et al., 2021; Romualdez, Walker, & Remington, 2021). In turn, many *camouflage* (i.e. hide) autistic characteristics to appear more neurotypical at work (Hull et al., 2017). Autism-specific initiatives may therefore not be an option for autistic people who experience high levels of self-stigma. Additional support about managing stigma (e.g. Han, Scior, Heath, et al., 2023; Han, Scior, Umagami, et al., 2023) may be a beneficial ‘bridge’ to enable engagement with employment programmes for individuals who are less comfortable disclosing their diagnosis. More broadly, however, employers must work hard to create safe and supportive working environments where autistic people can be free from stigmatisation and discrimination. Such efforts could contribute to an ultimate end goal where autism-specific schemes are not needed and disclosure is unnecessary because work environments are suitably understanding, accepting and supportive of neurodiversity.

Participants in the current study recognised that the EAN broke down barriers to employment by providing autism-specific support to interns and employers throughout the process. In particular, employers’ lack of understanding about autism, and miscommunications between employers and autistic employees, have been highlighted as barriers to sustained and fulfilling employment for autistic people (Black et al., 2020; Romualdez, Heasman, et al., 2021). While miscommunication was identified as an issue in the qualitative evaluations of PS + AS and DB UK (Remington et al., 2022; Remington & Pellicano, 2019; Romualdez et al., 2020), findings from the current study centre on enhanced two-way understanding between the interns and employers. This finding may be due to employers receiving training that has been shown to influence autism knowledge and commitment to inclusion in the workplace (Ashworth et al., 2023), and employers made use of the continued support, expertise and liaison services provided by the EAN throughout the internship. These elements likely address the double empathy problem (i.e. a lack of reciprocity between autistic and non-autistic people’s understanding of each other; Milton, 2012) that commonly affects the experiences of autistic people in the workplace (Milton et al., 2020).

This present study is one of the first to represent parental voices alongside those of autistic young adults and employers (see also Lee et al., 2019). Parents play a key role in providing support and advocating as their young person transitions to adulthood. Furthermore, those without parental support notice a stark disadvantage in the services afforded to them (Crane et al., 2022). Parents report anxiety about their young person’s future and a desire for independence, as demonstrated in the current study and others (e.g. Chen et al., 2019; Cribb et al., 2019). The current findings mapped onto parents’ reports in Lee et al.’s (2019) study that the work experience provided the parents with hope for their young person’s future. Drawing from this context, parents’ endorsement of the EAN as path to independence is an important endorsement of the utility and value of the initiative, and suggests its framework supports long-term benefits for the young adults and their transition into adulthood. In addition, the focused and continued support afforded by the EAN may remove sole reliance on parental support through the employment trajectory and increase accessibility to employment for autistic young adults without parental support.

Although views of the programme were overwhelmingly positive, interns, employers and parents all raised concerns related to some unmet expectations of the internship. These concerns included interns having different expectations of the work they would be doing, mismatch of skills to work, and employers having ambiguous or misjudged expectations of their interns’ abilities and preferences. In line with the disability rights movement’s philosophy *nothing about us without us* (Charlton, 1998), these insights highlight why it is so important to include autistic voices in evaluations of supported employment initiatives. Indeed, participants’ different – and in some cases – unmet expectations raised in the present study underline the need for multi-informant qualitative research. Interestingly, concerns regarding a mismatch between interns’ skills and demands of the job role were echoed in previous research into autism-specific internship schemes (Remington et al., 2022). As suggested by participants in the present study, this mismatch could be addressed by ensuring that recruitment processes involve sharing more detailed information in a ‘candidate profile’ about the intern’s work-related preferences and skillset.

The current study is not without its limitations. First, the current findings relate to a work experience initiative for autistic people without ID, so may not be applicable or appropriate for autistic people with ID. Nevertheless, there are similarities in qualitative themes between the current study and Romualdez et al.’s (2020) study with autistic young people with ID, such as increased confidence. Second, only 17.4% of possible interns engaged in an interview, so the current participant sample may not be representative of the all the interns engaging with EAN internships. This may be due to a self-selection bias whereby only participants who were more comfortable engaging with research and/or those who had relatively positive experiences of the EAN were more likely to volunteer to interview. Similarly, employers may have demonstrated social desirability bias in an effort to promote their organisation in a professional capacity and left out the more negative experiences of the internship, and/or may have already had more favourable views towards autism and neurodiversity compared with other organisations as they volunteered to be involved in the scheme. However, the multi-informant study design helps ensure better reliability of findings by highlighting areas of consistency and inconsistency between participant groups’ reports. Finally, there is little evidence about how employers’ rethinking of current practice and systems translated into behavioural or policy change at the workplace. This may be because the interviews were conducted soon after the internships finished so there was not enough time to implement any changes. Further research should follow-up with employers to assess the wider impact of the internship on aspects such as recruitment policies. Finally, it is important to examine the long-term outcomes of EAN internships, to assess their sustained impact for those involved.

## Conclusion

Our study provides the first multi-informant qualitative analysis of interns’, employers’, and parents’ experiences of a specific supported employment scheme for autistic young adults, the EAN. Our findings provide valuable evidence about one of the few supported employment work experience initiatives available to autistic young adults without ID, and offer useful insights into best practice. Results suggest that other supported employment initiatives for autistic people without ID should: (1) include a work experience component instead of focusing solely on skills, (2) address known autism-specific employment barriers such as adjusting application processes and improving employers’ autism understanding and acceptance and (3) invest in tailored support for the intern before, during and after the work experience to support long-term independence. Crucially, autistic people’s voices should be included in further evaluations and developments of future initiatives to ensure they are useful and appropriate for the autistic community.

## Supplemental Material

sj-docx-1-aut-10.1177_13623613231214834 – Supplemental material for Evaluating a new supported employment internship programme for autistic young adults without intellectual disability Supplemental material, sj-docx-1-aut-10.1177_13623613231214834 for Evaluating a new supported employment internship programme for autistic young adults without intellectual disability by Maria Ashworth, Brett Heasman, Laura Crane and Anna Remington in Autism

sj-docx-2-aut-10.1177_13623613231214834 – Supplemental material for Evaluating a new supported employment internship programme for autistic young adults without intellectual disability Supplemental material, sj-docx-2-aut-10.1177_13623613231214834 for Evaluating a new supported employment internship programme for autistic young adults without intellectual disability by Maria Ashworth, Brett Heasman, Laura Crane and Anna Remington in Autism

sj-docx-3-aut-10.1177_13623613231214834 – Supplemental material for Evaluating a new supported employment internship programme for autistic young adults without intellectual disability Supplemental material, sj-docx-3-aut-10.1177_13623613231214834 for Evaluating a new supported employment internship programme for autistic young adults without intellectual disability by Maria Ashworth, Brett Heasman, Laura Crane and Anna Remington in Autism

sj-docx-4-aut-10.1177_13623613231214834 – Supplemental material for Evaluating a new supported employment internship programme for autistic young adults without intellectual disability Supplemental material, sj-docx-4-aut-10.1177_13623613231214834 for Evaluating a new supported employment internship programme for autistic young adults without intellectual disability by Maria Ashworth, Brett Heasman, Laura Crane and Anna Remington in Autism

## Notes

1. While there is a strand of the Employ Autism network (EAN) that offers ‘Supported Internships’ for autistic individuals with intellectual disability (ID), the current study evaluated EAN internships for autistic adults without ID.

2. Note that the 19 intern participants who engaged in interviews constituted 17.4% of the 109 individual internship places offered by 12 organisations in 1 year of internships. The median number of internship places offered by the organisations was 1.5 (*M* = 9.1; *SD* = 23), but one organisation offered 85 internship places.

3. Quotes followed by a bracketed I, E, or P are from an intern, employer, or parent participant, respectively.
