# Supplementary material for: Evaluating a new supported employment internship programme for autistic young adults without intellectual disability
Source: Autism. 2023 Nov 28;28(8):1934–46. doi: 10.1177/13623613231214834 (PMC11301954; doi:10.1177/13623613231214834)
Supplement: sj-docx-1-aut-10.1177_13623613231214834 – Supplemental material for Evaluating a new supported employment internship programme for autistic young adults without intellectual disability [file sj-docx-1-aut-10.1177_13623613231214834.docx]

**Supplemental Material A**

**Table of stages of the [internship scheme] stages for (potential) interns and employers.**

| **Stage** | **Intern** | **Employer** |
| --- | --- | --- |
| **Recruiting organisations to [internship scheme]** | - Not applicable. | - [Charity] promotes the [internship scheme] via various marketing and networking strategies both online and in person events with potential host organisations. - The organisation volunteers to join the [internship scheme], and begins liaising with staff at [charity] to setup the internship. |
| **Pre-application** | - Roles are advertised on the [charity] website, and sent to those signed up to the [internship scheme] network mailing list. | - Employers attend online training hosted by [charity], [training name] *Understanding Autism in the Workplace*, that aims to equip employers with knowledge about autism and provide practical ways to support their interns and make their workplace more inclusive. |
|  | - Questions about the role and application process can be emailed to a specific email address and someone from the [charity] team can help. |  |
|  | - An optional pre-application meeting can be arranged with someone the team to discuss suitability to the role, and/or get advice on CVs and the application task. |  |
| **Application** | - The [charity] team is available for any questions regarding the application form and information about the application task. | - Employers are offered tailored resources, support, and expertise from the team at [charity] to make the interview process and any application task appropriate and accessible. |
|  | - All applicants receive feedback on their application whether they are successful or not, and unsuccessful applicants are offered a meeting with a careers advisor to discuss the feedback. |  |
| **Interview** | - For candidates offered an interview, the [charity] team can help with preparation in-person or virtually. | - Employers share interview questions with candidates, interview the candidates, review the quality of any tasks set for the application, and decide on the successful candidate/s. |
|  | - Interview questions and one-page profiles of the interviewer are shared with the candidate ahead of the interview. |  |
|  | - The candidate meets with a member of the team to develop a ‘candidate profile’, a document shared with the line manager and other interviewer that highlights strengths, interests, and any workplace adjustments required. |  |
| **During the internship** | - There is a pre-placement call a week before the internship starts to ensure the young person has everything they need and to answer any questions. | - Employers provide workplace adjustments with the support of staff at [charity] who can help the employers implement the adjustment appropriately. Examples of workplace adjustments include changes to working hours, logistics (e.g., how to travel into the office), communication preferences (e.g., written versus oral communication), reduced social obligation, changes to equipment, changes to the role, changes to clothing or appearance, the physical environment, flexible working location, or changes to supports (e.g., information resources, mentors). - The team at [charity] are available throughout the internship to offer support by answering questions from employers or providing relevant advice or information. |
|  | - The team at [charity] are available for questions or extra assistance for the onboarding process, for regular (but not mandatory) check ins with the intern throughout the placement, and can assist with requesting workplace adjustments or liaising other issues the young people are not comfortable discussing with their line manager. | - The [charity] team can also act as a liaison between the intern and the employer/s for any issues, such as requesting or implementing workplace adjustments. |
|  | - At the end of the internship, there is a final check-in with the team from [charity] to review the time on the internship. |  |
| **After the internship** | - The young people are offered access to careers meeting with a careers’ advisor. |  |
