# Supplementary material for: Evaluating a new supported employment internship programme for autistic young adults without intellectual disability
Source: Autism. 2023 Nov 28;28(8):1934–46. doi: 10.1177/13623613231214834 (PMC11301954; doi:10.1177/13623613231214834)
Supplement: sj-docx-2-aut-10.1177_13623613231214834 – Supplemental material for Evaluating a new supported employment internship programme for autistic young adults without intellectual disability [file sj-docx-2-aut-10.1177_13623613231214834.docx]

**Supplemental Material B**

**Interns’, Employers’, and Parents’ interview schedules.**

**Intern interview schedule**

## **Question Section 1: About you and your experiences so far.**

To start, I’d like to learn a little more about you and your experience of transitioning into adulthood so far. We will speak about the [internship scheme] in the next question section.

1. **Other than the [internship scheme] you have just completed, have you had any employment experiences?**

- Have you had any paid full-time or part-time jobs (e.g., working in a shop, cutting grass, or working in an office), volunteered, or done any internships?
  1. **If so, what were those employment experiences?**
  2. **What were those employment experiences like?**
     - Were they negative, positive or mixed? Why?

1. **Have you experienced any barriers that stopped you getting work?**
2. **Have you received any information or support about leaving education and finding/getting work experience?**

- Was there anyone or anything that helped you find and/or get work? For example, did anyone at school or university talk to you about work?
  1. **Was there anything that was particularly useful? If so, why?**
  2. **When you left education, was there any other information or support you would like to have had?**

1. **What area/areas of work are you interested in getting into?**
   - - What do you want to do for employment in the future?
     - What is your ideal career?

**Question Section 2: The [internship scheme]**

In this section I will ask you about your experiences of the [internship scheme].

1. **Please tell me about your internship.**
   1. **What organisation did you work for?**
   2. **What was your job title and role?**
      - What were your responsibilities?
      - What did you do day to day?
2. **What was the application process like for the [internship scheme]?**
   - - How it easy to access and complete?
     - Did anyone help you with the application?
3. **For this question, I’d like us to think back to before you did the internship.** **Why did you apply for the internship?**
   - - What skills or experience did you hope to get from the [internship scheme]?
   1. **Were you surprised by anything on the internship?**
      - Was there anything on the internship that was different to what you had expected?
4. **Now that you have done the internship, please tell me about how it felt to do the [internship scheme].**
   1. **Tell me about any positive or good experiences.**
      - What would you have kept the same?
   2. **Tell me about any challenging experiences.**
      - What would you have changed?
5. **What was the best thing you have gained from your [internship scheme]?**

*** Only ask if the individual has had previous work experience:*

1. **What was the [internship scheme] like in comparison to other work experience you have had in the past?**

- What was different?
- What was similar?

**Question Section 3: Looking ahead**

In this final section, I will ask some questions about your future.

1. **What are your plans for the future?**
   - Work? More education?
   - Has the [internship scheme] affected these plans in any way? (Do you feel it has prepared you for work life?)
2. **Is there anything else you’d like to say about the transition from leaving education and moving into work or adulthood, or the [internship scheme] that we haven’t talked about?**

**Employer interview schedule**

**Question Section 1: Before the [internship scheme]**

To start, I would like to learn a little more about you and ask you some questions about your views before the internship started. We will speak about the [internship scheme] in the next question section.

1. **Without naming people or places/organisations, have you ever *knowingly* worked with autistic people before this experience?**

**If yes,**

- 1. **Please tell me a bit about this. What was it like?**

1. **Did you attend the [internship scheme] training?**

**If yes,**

- 1. **What did you think of the training?**
     - What did you like about the training?
     - What didn’t you like about the training?
  2. **How did the training prepare you for welcoming an autistic young person as an intern at your workplace?**
  3. **Did you think anything was missing from the training?**

**Question Section 2: The [internship scheme]**

In this section I will ask you about your experiences of the [internship scheme].

1. **Did you work with the autistic young person/people doing the internships at your workplace?**
   1. **If yes, in what capacity did you work and interact with the autistic young person?**
2. **What were your expectations of the [internship scheme] and the internship?**
   - - What did you think it would be like?
   1. **Did the programme/internship meet your expectations?**
   2. **Were you surprised by anything on the internship?**
3. **How did the [internship scheme] impact your ability to work with the autistic young person/people?**
   - Did you feel like you had enough knowledge or support yourself to adequately support the autistic young person/people doing the internship?
4. **What were the benefits of providing an internship to the autistic young person/people at your workplace?**

- How was/were the young person/people an asset to the workplace?

1. **What were some difficulties or challenges in providing an internship to the autistic young person/people?**
   1. **How did you overcome (or try to overcome) these difficulties or challenges?**

**Question Section 3: Looking ahead**

In this final section I will ask some questions about your perspectives now that the internship has finished.

1. **If you had more autistic interns at your workplace, what would you do differently?**
2. **How has the [internship scheme]/internship affected your views, attitudes and awareness about autism?**

- What are your thoughts on working with more autistic people in the future?
  - Do you think your views on working with autistic people have changed? If so, how?

1. **How has this experience affected other aspects of your life?**
   - For example, how you interact and work with other colleagues, family or friends.
2. **What would you say to other organisations or colleagues about hiring autistic people?**
3. **Is there anything else you’d like to say about the working with the autistic young person/young people or the [internship scheme] that we haven’t talked about?**

**Parent interview schedule**

## **Question Section 1: Experiences so far**

To start, I’d like to learn a little more about your experience as a parent/carer of an autistic young person transitioning into adulthood so far. We will speak about the [internship scheme] in the next question section.

1. **What are your thoughts on the process of your young person finding and getting work experience?**
   - Was there information and/or support available for your young person to get relevant work experience?
   - Were there any barriers that stopped your young person getting work? Any enablers?
   - How involved have **you** been in these processes?
2. **Other than the [internship scheme], has your young person had any other work experience?**

**If yes,**

1. **What are your thoughts on previous work experiences your young person has had?**
   - What were those employment experiences like? Were they negative, positive or mixed? Why?
2. **If applicable, what information or support have you received as a parent/carer about your young person’s transition from education into work?**
   - How easy was it to access this support?
3. **Have you received any particularly useful support about your young person transitioning into adulthood and finding/getting work experience? Why was it useful?**
4. **Was there anything else you would have liked to have known about or had as someone supporting their young person transition from education into work?**

## **Question Section 2: The [internship scheme]**

In the next section I will ask you about your views and perspectives of the [internship scheme] and internship that your young person has been involved in.

1. **How did you find out about the [internship scheme]?**
   - - Did you or your young person know about it first?
2. **What did you hope your young person would get out of the [internship scheme] and internship?**
   - - What skills or experience did you hope your young person would get from the [internship scheme]?
     1. **Do you think your young person got what you were hoping they would get from the internship?**
     2. **Were you surprised by anything on the internship?**
3. **What do you think are the main benefits of your young person being involved in the [internship scheme]?**
4. **Did you have/have you any concerns about any aspect of the [internship scheme]?**
   1. **If so, have these concerns been alleviated?**

*** Only ask if the individual has had previous work experience:*

1. **If applicable, what was the [internship scheme] like in comparison to other work experience your young person has had in the past?**

- What was different?
- What was similar?

**Question Section 3: The future**

In this final section I will ask some questions about your views and perspectives on how you see the future for you and your young person as they transition into adulthood.

1. **What do you think your young person has gained from the [internship scheme]?**
2. **How has the [internship scheme] impacted or affected you as a parent or carer, if at all?**
   - How do you feel about your outlook on your young person’s future as they move into adulthood? Do you feel optimistic or concerned?
3. **What are your hopes and dreams for your young person as an adult?**
   - What achievements made by your young person would please you?
4. **How do you see yourself being involved in your young person’s transition into adulthood from now?**
   - What support would you like to have going forward?
   - What support would you like your young person to have going forward/?
5. **Is there anything else you’d like to say about the transition into work/adulthood, or the [internship scheme] that we haven’t talked about?**
