# Supplementary material for: Evaluating a new supported employment internship programme for autistic young adults without intellectual disability
Source: Autism. 2023 Nov 28;28(8):1934–46. doi: 10.1177/13623613231214834 (PMC11301954; doi:10.1177/13623613231214834)
Supplement: sj-docx-3-aut-10.1177_13623613231214834 – Supplemental material for Evaluating a new supported employment internship programme for autistic young adults without intellectual disability [file sj-docx-3-aut-10.1177_13623613231214834.docx]

**Supplemental Material C**

**Table of Themes and Illustrative Quotes**

*Quotes followed by a bracketed I, E, or P are from an intern, employer, or parent participant, respectively.*

| **Themes** | **Sub-themes** | **Illustrative Quotes** |
| --- | --- | --- |
| 1. "An invaluable experience" | *1.1. An opportunity for interns' self-development* | I wish I’d done it sooner really… maybe if I’d done it earlier, I would have felt more confident and more like I could do real jobs and I’d have applied for different things and I’d have been better prepared in interviews I’d done. (I5) |
|  |  | Knowing that they’re capable of something at this level, not to sell themselves short, and to gain a greater deal of self-awareness as to what might be challenging and what they can take in their stride. (P9) |
|  |  | I hope it's been a benefit for them… the feedback has been really, really great from everyone so I'm hoping [it's been] a bit of a confidence boost [for them] and [has provided] the opportunity that if they want to go elsewhere, they can because they can use us as a reference. (E22) |
|  | *1.2. A path to independence* | Having more self-assertion of my own decisions I think also giving my self-confidence with organising my day. (I16) |
|  |  | I like the independence, it was much less stressful and much more hands off than school. (I2) |
|  |  | Like any parents you just want your child to be happy and fulfilled. And in [our young person’s] case, I think just a bit more independence from myself and my husband, because we won’t be around forever. (P6) |
| 2. Breaking down barriers | *2.1. A way to get into employment* | The big corporate companies… they’re quite daunting when you’re on the outside but once you find your way in, like the [internship scheme] programme has helped me to do, there’s just so many opportunities to learn. (I3) |
|  |  | I didn’t think there was any support out there, so to come to [the [internship scheme] network] and people are trying to get the handle on how to get all these young people into work to make them feel valued [makes me feel confident]. (P3) |
|  | *2.2. Enhanced two-way understanding* | I think because the employers know they’re working with autistic interns, they know what to expect, maybe they’re going to have to offer some sort of flexibility or be more understanding… there was a lot more framework in place if I did need help, so I felt supported already. (I4) |
|  |  | Positive outcomes for the young people, positive outcomes for our organisation. (E4) |
|  |  | [There was] the massive impact on a colleague [being] able to divulge their neurodivergence. (E11) |
|  | *2.3. Challenging preconceptions and stereotypes* | I just made this assumption that during meetings [they] would want to be off video and off audio… and that wasn’t the case. [They] wanted to be on video, and [they] wanted everyone else to be on video as well. That kind of reinforced for me that you can’t make assumptions… that was definitely a key learning point. (E15) |
|  | *2.4. Rethinking current systems and structures* | I suppose [the internship experience] solidified, what can we do? What opportunities can we give individuals and not shy away if when we do our applications and [ask] are there any disability factors? We can actually put something to make sure we give individuals reasonable adjustments. (E8) |
|  |  | I would also encourage research and time to go into hiring an autistic person before, because what I think is quite unfair is if an autistic person was hired and were kind of used as a guinea pig to [see] how does an autistic person fit into our workplace. (E19) |
| 3. Differing expectations |  | A lot of people were nice, but then they would get too hyper conscious of overwhelming me and I get why but I personally find it a bit condescending and infantilising. (I15) |
|  |  | What I was given didn’t really make very clear what their capacity was or what they would be capable of doing, what they would be interested in doing. (E12) |
|  |  | Whether the [internship scheme] creates false comfort for the future… we don't really know yet. (P10) |
