# Supplementary material for: Evaluating a new supported employment internship programme for autistic young adults without intellectual disability
Source: Autism. 2023 Nov 28;28(8):1934–46. doi: 10.1177/13623613231214834 (PMC11301954; doi:10.1177/13623613231214834)
Supplement: sj-docx-4-aut-10.1177_13623613231214834 – Supplemental material for Evaluating a new supported employment internship programme for autistic young adults without intellectual disability [file sj-docx-4-aut-10.1177_13623613231214834.docx]

| ICMJE DISCLOSURE FORM | |
| --- | --- |
| **Date:** | 4/20/2023 |
| **Your Name:** | Maria Ashworth |
| **Manuscript Title:** | Evaluating a New Supported Employment Internship Programme for Autistic Young Adults Without Intellectual Disability |
| **Manuscript Number (if known):** | Click or tap here to enter text. |
| In the interest of transparency, we ask you to disclose all relationships/activities/interests listed below that are related to the content of your manuscript. “Related” means any relation with for-profit or not-for-profit third parties whose interests may be affected by the content of the manuscript. Disclosure represents a commitment to transparency and does not necessarily indicate a bias. If you are in doubt about whether to list a relationship/activity/interest, it is preferable that you do so.  The author’s relationships/activities/interests should be defined broadly. For example, if your manuscript pertains to the epidemiology of hypertension, you should declare all relationships with manufacturers of antihypertensive medication, even if that medication is not mentioned in the manuscript.  In item #1 below, report all support for the work reported in this manuscript without time limit. For all other items, the time frame for disclosure is the past 36 months. | |

|  | | | **Name all entities with whom you have this relationship or indicate none (add rows as needed)** | **Specifications/Comments (e.g., if payments were made to you or to your institution)** |
| --- | --- | --- | --- | --- |
| **Time frame: Since the initial planning of the work** | | | | |
| **1** | All support for the present manuscript (e.g., funding, provision of study materials, medical writing, article processing charges, etc.)  **No time limit for this item.** | | \|  \| **None** \| \| --- \| --- \|  \| Ambitious about Autism (AaA) \| Ambitious about Autism is the UK’s autism education and employment charity who developed and run the supported employment initiative, the Employ Autism network. Ambitious about Autism funded a PhD project (including tuition fees and maintenance stipend) to conduct an independent evaluation of the Employ Autism network. The PhD student and supervisors are based at IOE, UCL’s Faculty of Education and Society, and the findings from the current study are from the PhD project funded by AaA.  Payments were made to the institution IOE, UCL via the charity, Autistica, under grant agreement no. 7275. \| \| --- \| --- \| \| Autistica \| The UK autism research charity, Autistica, are sub-granters of this funding as the PhD project is related to a wider project originally funded by Autistica called Discover Autism Research and Employment (DARE). \| \|  \| Click the tab key to add additional rows. \| | |
| **Time frame: past 36 months** | | | | |
| **2** | | Grants or contracts from any entity (if not indicated in item #1 above). | \|  \| **None** \| \| --- \| --- \|  \|  \|  \| \| --- \| --- \| \|  \|  \| \|  \|  \| | |
| **3** | | Royalties or licenses | \|  \| **None** \| \| --- \| --- \|  \|  \|  \| \| --- \| --- \| \|  \|  \| \|  \|  \| | |
| **4** | | Consulting fees | \|  \| **None** \| \| --- \| --- \|  \| Ambitious about Autism (AaA) \| Ambitious about Autism provided consultation fees for two autistic young people to provide feedback on interview schedules for the current study. The fees were paid directly to the consultants from AaA. \| \| --- \| --- \| \|  \|  \| \|  \|  \| \|  \|  \| | |
| **5** | | Payment or honoraria for lectures, presentations, speakers bureaus, manuscript writing or educational events | \|  \| **None** \| \| --- \| --- \|  \|  \|  \| \| --- \| --- \| \|  \|  \| \|  \|  \| | |
| **6** | | Payment for expert testimony | \|  \| **None** \| \| --- \| --- \|  \|  \|  \| \| --- \| --- \| \|  \|  \| \|  \|  \| | |
| **7** | | Support for attending meetings and/or travel | \|  \| **None** \| \| --- \| --- \|  \| Ambitious about Autism (AaA) \| Part of the funding from AaA was for attending conferences (e.g., registration fees) and travelling to and from conferences. These payments were paid to the institution as part of the overall grant, and were claimed and reimbursed via the institutions’ HR expenses policies. \| \| --- \| --- \| \|  \|  \| \|  \|  \| | |
| **8** | | Patents planned, issued or pending | \|  \| **None** \| \| --- \| --- \|  \|  \|  \| \| --- \| --- \| \|  \|  \| \|  \|  \| | |
| **9** | | Participation on a Data Safety Monitoring Board or Advisory Board | \|  \| **None** \| \| --- \| --- \|  \|  \|  \| \| --- \| --- \| \|  \|  \| \|  \|  \| | |
| **10** | | Leadership or fiduciary role in other board, society, committee or advocacy group, paid or unpaid | \|  \| **None** \| \| --- \| --- \|  \|  \|  \| \| --- \| --- \| \|  \|  \| \|  \|  \| | |
| **11** | | Stock or stock options | \|  \| **None** \| \| --- \| --- \|  \|  \|  \| \| --- \| --- \| \|  \|  \| \|  \|  \| | |
| **12** | | Receipt of equipment, materials, drugs, medical writing, gifts or other services | \|  \| **None** \| \| --- \| --- \|  \|  \|  \| \| --- \| --- \| \|  \|  \| \|  \|  \| | |
| **13** | | Other financial or non-financial interests | \|  \| **None** \| \| --- \| --- \|  \|  \|  \| \| --- \| --- \| \|  \|  \| \|  \|  \| | |
|  | |  |  | |
| **Please place an “X” next to the following statement to indicate your agreement:** | | | | |
|  | | I certify that I have answered every question and have not altered the wording of any of the questions on this form. | | |
